# Acute HIV infection syndrome mimicking COVID-19 vaccination side effects: a case report

**DOI:** 10.1186/s12981-021-00407-2

**Published:** 2021-10-26

**Authors:** Julian Triebelhorn, Stefanie Haschka, Felix Hesse, Johanna Erber, Simon Weidlich, Marcel Lee, Dieter Hoffmann, Josef Eberle, Christoph D. Spinner

**Affiliations:** 1 Department of Internal Medicine II, University Hospital Rechts Der Isar, School of Medicine, Technical University of Munich, Ismaninger-Straße 22, 93675 Munich, Germany; 2 Institute of Virology, School of Medicine, Technical University of Munich, Munich, Germany; 3 Max Von Pettenkofer Institute, German National Reference Centre for Retroviruses at Ludwig Maximilian University of München, Munich, Germany

**Keywords:** Acute HIV infection, COVID-19, Vaccination, HIV seroconversion illness, Fiebig, Seroconversion

## Abstract

**Background:**

Symptoms of primary HIV infection, including fever, rash, and headache, are nonspecific and are often described as flu-like. COVID-19 vaccination side effects, such as fever, which occur in up to 10% of people following COVID-19 vaccination, can make the diagnosis of acute HIV infection even more challenging.

**Case presentation:**

A 26-year-old man presented with fever and headache following COVID-19 vaccination. The symptoms were initially thought to be vaccine side effects. A diagnostic workup was conducted due to persisting fever and headache > 72 h following vaccination, and he was diagnosed with Fiebig stage II acute HIV infection, 3 weeks after having unprotected anal intercourse with another man.

**Conclusion:**

Thorough anamnesis is key to estimating the individual risk of primary HIV infection, in patients presenting with flu-like symptoms. Early diagnosis and initiation of antiretroviral therapy is associated with better prognosis and limits transmission of the disease.

## Background

HIV seroconversion describes the initial immune response of developing human immunodeficiency virus (HIV)-specific antibodies, which typically become detectable 22–24 days after infection (Table 1). Fiebig et al. [1] subdivided the process of HIV seroconversion into six stages, depending on the antibody–antigen pattern and HIV viral load in the blood. This classification allows a precise description of patients with primary HIV infection. In 89% of cases, the process of seroconversion is accompanied by symptoms, known as HIV seroconversion illness [2]. Symptoms often include malaise, headache, fever, generalized lymphadenopathy, diarrhea, and rash. In severe cases patients may experience meningeal symptoms that require hospitalization. The common symptoms of HIV seroconversion are nonspecific and are often described as flu-like, making it difficult to distinguish between HIV seroconversion illness and a wide range of other conditions, including vaccine side effects. During the COVID-19 mass vaccination period within the ongoing COVID-19 pandemic, vaccine side effects should be considered in the differential diagnosis, provided that the patient had recently been vaccinated. COVID-19 vaccine side effects include fatigue, redness, fever, arthralgia, and headache, and may resemble symptoms of HIV seroconversion illness, thus impeding the diagnosis of HIV [3].

**Table 1:** Stages of HIV markers/ diagnostic tests in early infection, as described by Fiebig et al.

Fiebig stage	HIV-PCR	4th-generation screening test	Western blot	p24- antigen	Antibodies	Days since infection
Eclipse	−	−	−	−	−	0–11
I	+	−	−	−	−	12–16
II	+	+	−	+	−	17–21
III	+	+	−	+	HIV-IgM: +	22–24
IV	+	+	inconclusive	+ / −	2/3 antibodies positive (p24, gp41, gp120)	25–30
V	+	+	+	+ / −	3/3 antibodies positive (p24, gp41, gp120) lacking p31	31–100
VI	+	+	+	+ / −	3/3 antibodies positive (p24, gp41, gp120), p31-antibodies positive	Open-ended

The table is as described by Fiebig et al., modified after [1, 4, 5]. Results of diagnostic tests (HIV-PCR, fourth-generation screening tests, Western blot) and detectability of HIV markers (p24-antigen, HIV-specific antibodies) are shown in relation to days since infection. (+) indicates positive test result/ detectability, (−) indicates negative test result/ HIV-marker not detectable. Eclipse and Fiebig stages are listed in dependence of HIV-marker, as described by Fiebig et al.

We describe a case of HIV seroconversion illness that was initially misdiagnosed as a COVID-19 vaccine side effect, to illustrate the similarities between common COVID-19 vaccination side effects and the diagnostic process needed to differentiate the two conditions.

## Case report

**Fig. 1:**
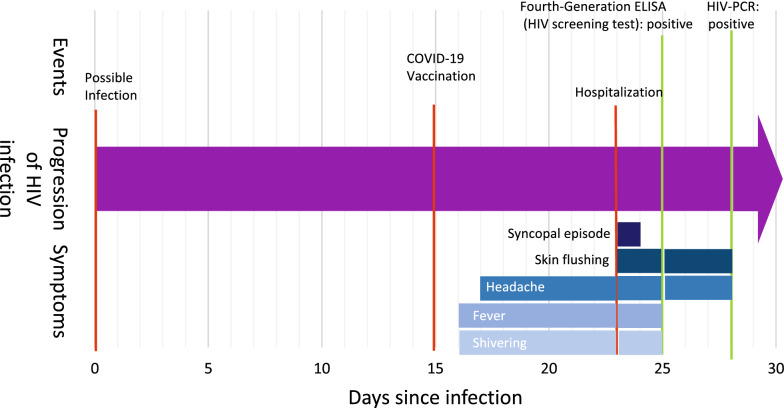
Timeline of the patient’s exposure history, clinical presentation, and diagnosis. Symptoms and events as described in this case report are depicted in a timeline measured in days since infection. Events are indicated by red bars, and positive test results are indicated by green bars. Symptoms and duration of persistence are illustrated by differently shaded blue bars

One day after receiving the first dose of COVID-19 vaccine with mRNA-1273 (Moderna Biotech, Cambridge, Massachusetts, USA) a 26-year-old German man developed fever, shivering and headache. During the following days, he developed rash, vertigo and nausea, culminating in a single syncopal episode 8 days after the vaccination, as shown in Fig. 1. The patient was referred to the nearest local hospital because his symptoms were suspected to be due to vaccine-associated side effects. He was provided with analgesics and fluid, and the symptoms rapidly resolved. Detailed sexual history-taking revealed an episode of unprotected anal intercourse with another man 23 days previously. He subsequently tested positive for HIV on a fourth-generation p24/ab-enzyme-linked immunosorbent assay (ELISA). The patient was referred to our tertiary medical center for further assessment and initiation of treatment.

On arrival, the patient complained of a mild headache, general weakness and persisting skin flushing. Blood biochemistry showed an elevated C-reactive protein (CRP) level (2.0 mg/dL; reference: < 0.5 mg/dL) and hematology showed lymphocytopenia (2.49 G/L), with a CD4 T-cell count of 284 cells/μL and a CD4/CD8 ratio of 0.2. He was retested for HIV using a screening test (HIV Ag/Ab Combo performed on Abbott Architect, Chicago, Illinois, USA) and HIV-immunoblot (recomLine HIV-1 and HIV-2 by Mikrogen, Neuried, Germany). The screening test was reactive but the HIV-specific western blot was negative and no HIV-specific antibodies were detected. Realtime HIV polymerase chain reaction (PCR) testing (Abbott, Chicago, Illinois, USA) revealed a high viral load of 49,817,530 copies/mL, thus confirming the diagnosis as Fiebig stage II acute HIV infection, as shown in Table 1. Coinfection with SARS-CoV-2 was excluded multiple times by SARS-CoV-2 PCR (TaqMan™-PCR performed on Roche cobas^®^ 6800, Basel, Swiss). The patient was discharged without any symptoms and initiated on antiretroviral therapy with bictegravir/emtricitabine/tenofovir alafenamide.

## Discussion and conclusion

This case report illustrates how symptoms of acute HIV infection can be mistaken for side effects of COVID-19 vaccination, especially during the current period of mass COVID-19 vaccination during the ongoing pandemic. Given the similarity of symptoms, including fatigue, fever, erythema, headache, and drowsiness, primary HIV infections can easily be overlooked, and symptoms of acute HIV infection can be falsely attributed to vaccine side effects. During the diagnostic evaluation, detailed history-taking, including inquiring about recent sexual behavior, is key to assessing the risk of HIV infection. Risk factors or indicators include unprotected sexual intercourse, the presence of other sexually transmitted infections, injection drug use, receiving injections with unsterile equipment, accidental needlestick injuries, and unsterile cutting or piercing. Especially in high-risk groups, such as men who have sex with men, sex workers, and injection drug users, patients reporting flu-like symptoms should always be questioned about their recent sexual behavior and drug use history to identify a history of possible HIV exposure and followed up by thorough diagnostic testing for recent HIV infection. Oral ulceration and weight loss have the highest specificity, while fever and rash are the best independent predictors of primary HIV infection, making these symptoms particularly important to note [6].

Although testing for HIV is very reliable in diagnosing infection due to the high sensitivity of HIV screening tests and high specificity of confirmatory testing according to predetermined guidelines, there are still diagnostic gaps in early infection [7]. Such gaps could easily lead to the diagnosis of acute HIV infection being missed, especially if the symptoms occur following vaccination. Though significant progress has been made in diagnostics for early diagnosis, the delay between infection and fourth-generation HIV screening tests becoming reactive is approximately 17–21 days (or 22–24 days with third-generation screening tests) (Table 1). It takes further 14 days until the reactive screening test can be confirmed to enable time for formation of enough HIV-specific antibodies to achieve the predetermined criteria (3/3 antibodies positive against: p24, gp41, gp120). These diagnostic gaps make it easy to abandon a preliminary diagnosis of primary HIV infection, if the screening test result is not confirmed by the initial confirmatory test.

The possible consequences of misdiagnosis of primary HIV infection are serious. By missing the opportunity for early diagnosis and treatment, infected individuals could experience progression of disease thus leading to a worse outcome. The START trial showed that early initiation of treatment lowers the risk of acquired immunodeficiency syndrome (AIDS) related events by 72% and of non-AIDS events by 39% [8]. Early initiation of antiretroviral therapy (ART) also limits the HIV reservoir and allows optimal immune restoration, thus enabling best possible outcome [9].

Furthermore, overlooking the diagnosis in the stage of primary HIV infection misses the opportunity to prevent HIV transmission to others. The concentration of HIV RNA is reached in early stages of infection around the time of seroconversion, making these individuals especially contagious. Brenner et al. [10] estimated that approximately half of the cases of HIV transmission occur in the early stage of infection, making early diagnosis and treatment a powerful tool in preventing the spread of HIV.

In conclusion, this case report highlights the possibility of symptoms and test results in early HIV infection being misinterpreted due to concurrent COVID-19 vaccination and the diagnostic gap in early infections, making it easy to falsely attribute symptoms to vaccine-associated side effects.

## Data Availability

All data generated or analyzed during this study are included in this published article.

## Abbreviations

AIDS: Acquired immunodeficiency syndrome
ART: Antiretroviral therapy
COVID-19: Coronavirus disease
CRP: C-reactive protein
ELISA: Enzyme-linked immunosorbent assay
HIV: Human immunodeficiency virus
MSM: Men who have sex with men
PCR: Polymerase chain reaction
